# How Community-Based Teams Use the Stroke Recovery in Motion Implementation Planner: Longitudinal Qualitative Field Test Study

**DOI:** 10.2196/37243

**Published:** 2022-07-29

**Authors:** Jessica Reszel, Joan van den Hoek, Tram Nguyen, Gayatri Aravind, Mark T Bayley, Marie-Louise Bird, Kate Edwards, Janice J Eng, Jennifer L Moore, Michelle L A Nelson, Michelle Ploughman, Julie Richardson, Nancy M Salbach, Ada Tang, Ian D Graham

**Affiliations:** 1 Ottawa Hospital Research Institute Ottawa, ON Canada; 2 School of Nursing University of Ottawa Ottawa, ON Canada; 3 School of Epidemiology and Public Health University of Ottawa Ottawa, ON Canada; 4 March of Dimes Canada Toronto, ON Canada; 5 Division of Physical Medicine and Rehabilitation University of Toronto Toronto, ON Canada; 6 The KITE Research Institute University Health Network Toronto, ON Canada; 7 College of Health and Medicine University of Tasmania Tasmania Australia; 8 Department of Physical Therapy University of British Columbia Vancouver, ON Canada; 9 South Eastern Norway Regional Knowledge Translation Center Sunnaas Rehabilitation Hospital Oslo Norway; 10 Institute for Knowledge Translation Carmel, IN United States; 11 Lunenfeld-Tanenbaum Research Institute Sinai Health Toronto, ON Canada; 12 Dalla Lana School of Public Health University of Toronto Toronto, ON Canada; 13 Faculty of Medicine Memorial University of Newfoundland St John's, NL Canada; 14 School of Rehabilitation Science McMaster University Hamilton, ON Canada; 15 Department of Physical Therapy University of Toronto Toronto, ON Canada

**Keywords:** knowledge translation, knowledge mobilization, implementation science, implementation planning, stroke, rehabilitation, capacity building, community-based exercise programs

## Abstract

**Background:**

The Stroke Recovery in Motion Implementation Planner guides teams through the process of planning for the implementation of community-based exercise programs for people with stroke, in alignment with implementation science frameworks.

**Objective:**

The purpose of this study was to conduct a field test with end users to describe how teams used the Planner in real-world conditions; describe the effects of Planner use on participants’ implementation-planning knowledge, attitudes, and activities; and identify factors influencing the use of the Planner.

**Methods:**

This field test study used a longitudinal qualitative design. We recruited teams across Canada who intended to implement a community-based exercise program for people with stroke in the next 6 to 12 months and were willing to use the Planner to guide their work. We completed semistructured interviews at the time of enrollment, monitoring calls every 1 to 2 months, and at the end of the study to learn about implementation-planning work completed and Planner use. The interviews were analyzed using conventional content analysis. Completed Planner steps were plotted onto a timeline for comparison across teams.

**Results:**

We enrolled 12 participants (program managers and coordinators, rehabilitation professionals, and fitness professionals) from 5 planning teams. The teams were enrolled in the study between 4 and 14 months, and we conducted 25 interviews. We observed that the teams worked through the planning process in diverse and nonlinear ways, adapted to their context. All teams provided examples of how using the Planner changed their implementation-planning knowledge (eg, knowing the steps), attitudes (eg, valuing community engagement), and activities (eg, hosting stakeholder meetings). We identified team, organizational, and broader contextual factors that hindered and facilitated uptake of the Planner. Participants shared valuable *tips from the field* to help future teams optimize use of the Planner.

**Conclusions:**

The Stroke Recovery in Motion Implementation Planner is an adaptable resource that may be used in diverse settings to plan community-based exercise programs for people with stroke. These findings may be informative to others who are developing resources to build the capacity of those working in community-based settings to implement new programs and practices. Future work is needed to monitor the use and understand the effect of using the Planner on exercise program implementation and sustainability.

## Introduction

### Background

With more people surviving stroke [1], community-based exercise programs [2-4] have emerged to provide safe and effective exercise opportunities for people living with the effects of stroke. Although participating in exercise after stroke conveys broad benefits [5], most people with stroke lack access to these specialized exercise programs in their own communities. As a result of this gap, the Canadian Partnership for Stroke Recovery identified increasing implementation and sustainability of these community-based exercise programs as a knowledge translation priority [6,7].

Planning and implementing new evidence-based practices and programs is a complex process requiring specific knowledge and skills. There is a growing body of literature and resources aimed at building the capacity for implementation science, but there is less support for building the capacity of those actually practicing implementation [8-10]. Practitioners may not have the knowledge or skills to apply implementation science theories or frameworks in their work [11,12], nor do they report feeling confident in their ability to complete key steps in implementation planning, such as conducting a barriers and facilitators assessment [12]. Community organizations in particular may face barriers to implementing evidence-based practices, including a lack of implementation expertise [13].

As a means to build implementation-planning capacity for community-based exercise programs for people with stroke, our team developed an implementation-planning guide, the Stroke Recovery in Motion Implementation Planner (hereafter referred to as the Planner) [14]. The Planner was based on established knowledge translation and implementation frameworks, including the Knowledge-to-Action framework [15], CAN-IMPLEMENT [16,17], and the Implementation Planning Roadmap [18]. The focus of the Planner is on implementation *planning*, which is a process that turns strategy into action through three phases: phase 1: establishing a diverse, interdisciplinary planning team and working together to understand the community population, their needs, potential program options, and the available resources; phase 2: conducting a barriers and drivers assessment and developing tailored solutions (implementation strategies), as well as building an evaluation plan; and phase 3: launching, monitoring, and maintaining the exercise program.

Many strategies promoting uptake of evidence-based practices focus on modifying the individual or their environment. Throughout the Planner development and evaluation process [7], we prioritized the design of the product itself by applying a user-centered design approach [19]. User-centered design is an iterative process whereby the needs and context of the intended users are central to informing the content and design of the product [19,20]. An example of a user-centered design strategy is to field test the product. Through prolonged engagement with end users who provide feedback on their experience with the product, field testing facilitates an understanding of how the product is being applied in real-world settings [21]. Such an exploratory approach can help to challenge the prototype and subsequently lead to meaningful changes [22], thereby creating a product that is more functional, acceptable, and effective. Upon completing a prototype of the Planner [7], we conducted a field test study with end users to explore how the Planner was used and adapted in diverse settings.

### Objectives

This study was part of a larger research program that developed and evaluated the Planner, which included a cross-sectional user evaluation of the Planner by diverse stakeholders (reported elsewhere [7]) and the field test reported here. The objectives of the field test were to (1) describe how teams used the Planner in real-world conditions; (2) describe the effects of using the Planner on participants’ implementation-planning knowledge, attitudes, and activities; and (3) identify factors influencing use of the Planner.

## Methods

### Design

This field test study used a longitudinal qualitative design [23,24], which facilitated in-depth discussions about use and impressions of the Planner over time. We used the Consolidated Criteria for Reporting Qualitative Research (COREQ) checklist [25] to inform the reporting of this study (Multimedia Appendix 1).

### Participants and Setting

Using the professional networks of the study team, we identified primary contacts (ie, team leads tasked with program planning) at Canadian community organizations that were intending to implement a community-based exercise program for people with stroke in the next 6 to 12 months (*current* planners) and willing to use the Planner to guide their ongoing planning. We welcomed representatives from several occupations, including program managers and coordinators, health partners (eg, physiotherapists and occupational therapists), and fitness professionals. As the purpose of this study was to observe how teams used the Planner to guide planning processes, teams were required to be at an early stage in their planning. A research team member (J Reszel) contacted potential participants by email with study information, with up to two reminders. If the contact was interested in participating, a memorandum of understanding was signed between the participating organization and our research institution. We asked the contact person to nominate other planning team members (ie, snowball sampling) whom we could invite to take part in the study. All participants signed an individual consent form. Participants did not receive any incentives to test the Planner or fund their program initiatives; however, participants were provided an honorarium to compensate them for time spent on study activities.

Initially, we aimed to enroll 9 to 12 teams to field test the Planner; however, the onset of the COVID-19 pandemic resulted in significant disruptions to community-based program planning and the closure of many facilities. This recruitment challenge caused us to lower our enrollment to 5 teams prepared to use the Planner to guide program planning during this period.

### Data Collection

The field test teams participated in 3 core data collection points. After reading the Planner and completing a questionnaire [7], all participants completed a *baseline* interview or focus group to discuss their initial impressions of the planning process. Next, research staff conducted *monitoring interviews* (ideally every 1-2 months) with a primary contact for each team to discuss planning work completed, Planner sections and tools used, and feedback on what was helpful and what was not. Each monitoring call started with the researcher summarizing the last call, allowing the participants to make corrections. Finally, primary contacts completed an *end-of-study* interview. At this time (February-March 2021), each team was at a different point in their planning process, but this end-of-study interview provided an opportunity for participants to reflect on their overall experience of using the Planner to date and share final feedback. The primary contact was the team member who was taking the lead in the planning process and had the most direct experience working through all Planner steps and activities. We anticipated that this person could therefore provide the richest updates on the team’s work throughout the process.

In keeping with a user-centered design approach, the baseline, monitoring, and end-of-study interview guides (Multimedia Appendix 2) focused on the users’ needs and their experiences and contexts as they engaged with the Planner [19,20]. The discussions were all conducted by video call or phone. The interviews were audio recorded, and the baseline and end-of-study interviews were transcribed verbatim. Field notes were written after the discussions to document observations about the setting and participants’ (including interactions between participants in focus groups) and interviewer’s reflections. All data collection was conducted by 1 cisgender female research coordinator (J Reszel), a master’s-prepared registered nurse experienced in qualitative research. At the start of the study, the researcher had no relationship with participants. Extensive notes were taken during each monitoring call and later verified and enhanced using the audio recording to complete the call log (Multimedia Appendix 3). In addition, we asked each team to share completed planning tools to understand how teams used and adapted the Planner material.

### Data Analysis

Applying a cross-sectional approach [23], we used conventional content analysis [26] to inductively code all transcripts and monitoring-interview notes as they became available. This allowed us to identify what the teams were currently working on and their perceptions of the Planner at that time. This approach facilitated probing and follow-up in the subsequent interviews with the participant. All interviews were coded in Microsoft Word by 1 research staff member (J Reszel), with 20% coded independently by a second research team member (TN) as a form of analyst triangulation to enhance credibility [27,28]. Any differences in coding were discussed and resolved by the 2 coders. As analysis progressed, coding and findings were discussed at regularly scheduled meetings with the core research team. We also discussed the field notes for additional context on the setting and team dynamics, such as the extent to which different participants contributed to the discussion and which questions they answered versus those they did not answer. As codes emerged from the transcripts and notes, and the coding scheme was developed, we grouped similar codes into broader categories, including contextual information, Planner feedback, the planning process, and how the Planner is used. We also analyzed the data temporally [23] to identify changes in perceptions over time and to create timelines to map if and when teams completed the various Planner activities. The completed Planner tools were reviewed by the research team and documented as completed or not. When reviewing the tools, the researchers assessed whether the teams made any adaptations to the tools and whether they were used as intended. Exemplary completed tools were identified to be included in the Planner, with permission.

### Ethics Approval

We received ethics approval for this study from the Ottawa Health Science Research Ethics Board (20190594-01H). Before starting any study procedure, each participant signed a consent form.

## Results

### Demographic and Contextual Information on Planning Teams

We enrolled 5 planning teams in Canada. The teams were diverse in their geography and composition. There were teams representing the west coast to east coast of Canada in urban and rural settings. The teams ranged from a single person leading all aspects of planning to interdisciplinary teams sharing the planning work (Table 1).

**Table 1:** Attributes of teams taking part in the field test study.

		Team 1	Team 2	Team 3	Team 4	Team 5
**Planning team information**						
	Geographic area of planning team	Western Canada	Western Canada	Atlantic Canada	Central Canada	Central Canada
	Number of people on core planning team identified at the time of study	4	1	6	4	2
	Occupations of planning team members	Physiotherapist; fitness coordinator; fitness professionals	Program coordinator	Physiotherapist; program coordinator; fitness professional; person with stroke	Physiotherapist; occupational therapist; rehabilitation manager	Program coordinators
	Multiorganization collaboration?	Yes (municipality and private physiotherapy practice)	No (municipality only)	Yes (municipality and health authority)	Initially: no (primary care center only); during study: yes (municipality and primary care center)	No (community-based nonprofit only)
	Types of partners participating^a^ in planning process	Brain injury group; municipality; physiotherapy clients; stroke club	Local university; health authority; local stroke association	Health authority; inpatient rehabilitation services; outpatient rehabilitation services; municipality	Allied health partners in clinic; clinic clients; municipality	Internal staff; past program participants
**Program information**						
	Planned geographic area for program implementation	City	City	City	City	National (web-based)
	Population density of community where program would be offered	Rural or mostly rural	Urban or mostly urban	Urban or mostly urban	Rural or mostly rural	Combination of urban and rural
	Size of community where program would be offered	10,000 to 24,999	>50,000	25,000 to 50,000	5000 to 9999	National
	Type of organization planning to offer program	Municipality	Municipality	Municipality	Family health team	Web-based

^a^Ranging from consultation to collaboration, as per the International Association for Public Participation (IAP2) Spectrum of Public Participation [29].

### Team Composition

To provide context for interpreting the findings, we provide a brief description of each planning team before describing the study participants.

Team 1 included a physiotherapist in private practice and a program coordinator from the municipality who had previously collaborated to plan and implement other adapted fitness programs in the community. On the basis of their previously successful partnership and the perceived need for stroke-specific community programs, they decided to plan a new program together.

Team 2 comprised a single program coordinator from the municipality who was working within its usual organizational model whereby an individual coordinator is largely responsible for all planning activities. The municipality had an existing suite of adapted fitness programs and wished to explore adding a stroke-specific exercise class to its model.

Team 3 was led by a physiotherapist who had previously piloted a stroke-specific exercise program in a long-term care setting. The municipality had expressed interest in collaborating with the health authority on an adapted exercise program. The physiotherapist subsequently formed a new partnership with the municipality to begin planning a stroke-specific exercise program in the community.

Team 4 comprised rehabilitation health professionals and a manager from a primary care clinic affiliated with the local hospital. The team members had experience planning and implementing other group programs tailored to various health conditions within their primary care setting and were considering offering a stroke-specific program in their clinic.

Team 5 included 2 program coordinators from a nonprofit organization planning a web-based adapted exercise program. Before the COVID-19 pandemic, the program was offered in person. The onset of the pandemic provided an opportunity to explore whether the program could transition to a new web-based format reaching a broader geographical area.

### Field Test Study Participants and Data Collected

From these 5 teams, we enrolled 12 participants. Between February 2020 and March 2021, we conducted 25 interviews and focus groups with the 12 participants: the 7 baseline sessions lasted an average of 57 (range 40-75) minutes, the 13 monitoring calls lasted an average of 27 (range 18-35) minutes, and the 5 end-of-study sessions lasted an average of 44 (range 31-53) minutes. On average, the planning teams were followed by the research team for 9.6 (range 4-14) months (Table 2).

**Table 2:** Data collection approaches and description of study participants (N=12).

				Team 1 (n=4)		Team 2 (n=1)		Team 3 (n=1)		Team 4 (n=4)		Team 5 (n=2)
**Data collection**												
	Dates of participation in study		December 2019 to February 2021 (14 months)^a^		April 2020 to March 2021 (11 months)		May 2020 to February 2021 (9 months)		June 2020 to March 2021 (9 months)		October 2020 to February 2021 (4 months)	
	Types of qualitative data collected		1 baseline interview and 1 baseline FG^b^ (with 3 participants); 1 monitoring call; 1 end-of-study interview		1 baseline interview; 3 monitoring calls; 1 end-of-study interview		1 baseline interview; 3 monitoring calls; 1 end-of-study interview		1 baseline interview and 1 baseline FG (with 3 participants); 4 monitoring calls (with 2 participants); 1 end-of-study FG (with 2 participants)		1 baseline FG (with 2 participants); 2 monitoring calls (with 2 participants); 1 end-of-study FG (with 2 participants)	
**Study participants’ role on the planning team, n (%)**												
	Program manager or coordinator		1 (25)		1 (100)		0		1 (25)		2 (100)	
	Rehabilitation health professional		1 (25)		0		1 (100)		3 (75)		0	
	Fitness professional		2 (50)		0		0		0		0	
**Study participants’ previous experience in *planning* adapted or specialized fitness programs, n (%)^c^**												
	Yes		2 (67)		1 (100)		1 (100)		3 (75)		1 (50)	
	No		1 (33)		0		0		1 (25)		1 (50)	
**Study participants’ previous experience in *delivering* adapted or specialized fitness programs, n (%)^c^**												
		Yes		2 (67)		1 (100)		1 (100)		4 (100)		1 (50)
		No		1 (33)		0		0		0		1 (50)

^a^We lost contact with team 1 between month 2 and month 11, both inclusive, and no data were collected during this time.

^b^FG: focus group.

^c^A participant from team 1 did not complete the questionnaire that included these demographic questions.

### How Teams Used the Planner in Real-world Conditions

#### Overview

**Figure 1:**
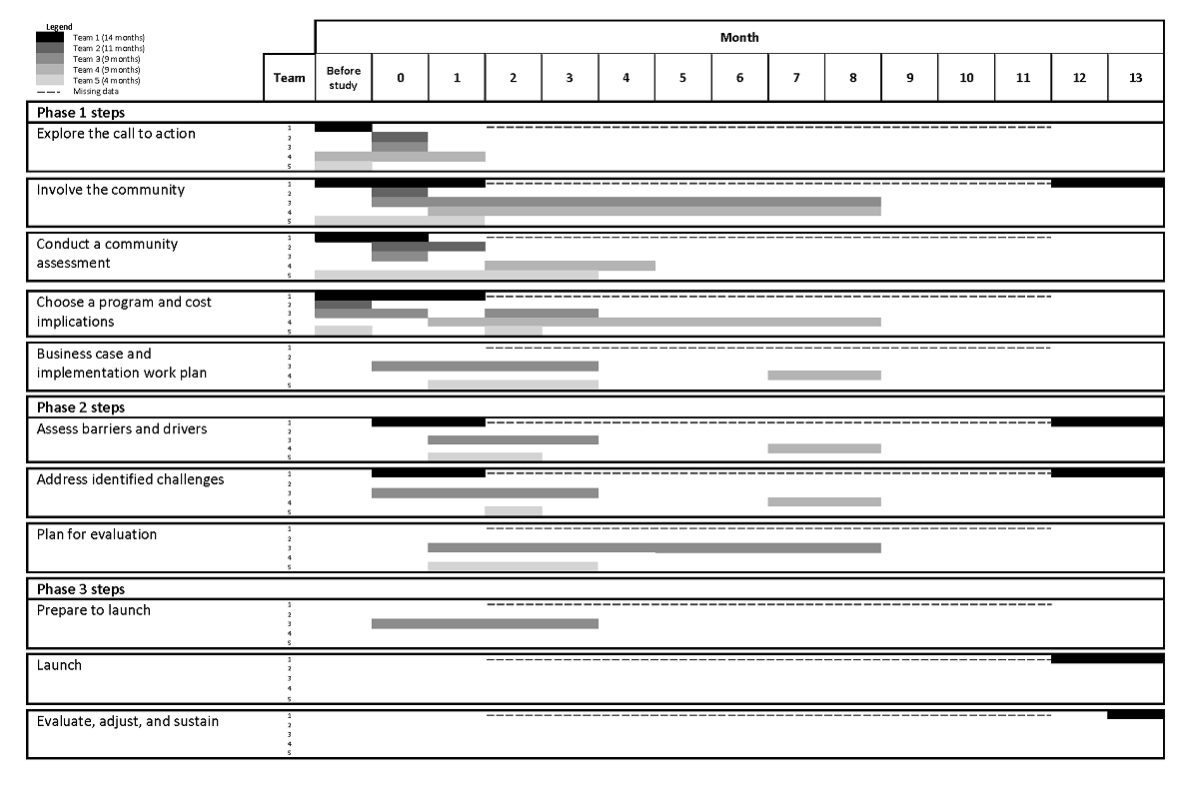
Summary of Planner phases and steps completed by field test teams during the study period.

The 5 teams varied in how they used the Planner, ranging from methodically working through each step to using the Planner more as a reference guide when needed. Figure 1 provides a visual summary of the phases and steps the teams completed during the study period. Most of their planning progress focused on phase 1. Teams took from 1 to ≥8 months to focus on the early planning activities. Because of the impact of the pandemic (eg, facility closures and suspended programs), the teams completed fewer activities beyond phase 1. Despite these challenges, of the 5 teams, 4 (80%) were able to complete at least one step in phase 2 or phase 3. Instead of moving through the steps sequentially, the teams tended to use a nonlinear approach to address many tasks concurrently and revisited some steps multiple times. The results are organized by implementation-planning phase and step.

#### Explore the Call to Action

The call to action was one of the first steps completed by each team. Although most (4/5, 80%) of the teams had previously been considering the value of planning a stroke-specific exercise program in their community, these teams described how participating in the field test prompted them to formally start their planning process.

#### Involve the Community

We observed that involving the community was the step that required the greatest amount of time (Figure 1), reflecting the need to engage community partners throughout the entire planning process. All teams had an intensive focus on involving the community at the front end as they identified key partners to join the planning team and relevant stakeholders to consult. Participants from all teams reported that using the Planner prompted them to consider alternative ways of identifying and engaging people or groups. For example, team 2 reconsidered their usual way of working independently within their own organization and began reaching out to stroke-related organizations in the community. Team 3 decided to add representatives from key referral sources as well as a person living with stroke and their caregiver to the planning team and explored the need for developing a formal partnership agreement. Involving these different partners informed referral pathways and how to accommodate participant needs. This team used the Planner itself as a tool to structure team meetings and engage team members:

I think it (the Planner) was quite good actually because it just allowed me to know where to focus and make some plans around how those meetings were going to look. It was really very helpful for meeting planning to focus from a team perspective. I think if it was just myself doing it I might have been able to manage without the Planner, but when you’re trying to actually organize your thoughts and articulate them to other people I think the Planner was really helpful in doing that.

Physiotherapist, team 3, ID6, end of study

#### Conduct a Community Assessment

The teams described several methods to conduct their community assessments, which were completed at various time points in the planning process. For example, based on an early assessment of services in the community and the number of people living with stroke, team 1 decided to move forward with planning and implementing a program. However, after launching the program and having lower than anticipated enrollment, this team appreciated the importance of taking the time to complete a detailed community needs assessment upfront:

Make sure there’s a need for it in your community...do that research before you start so that you’re not launching a program that you get only three registrants for, and you don’t know if you can run it after all that work.

Physiotherapist, team 1, ID1, end of study

At the start of the planning process, team 4 felt confident that they would offer a future program in their clinic setting. However, as they took inventory of the programs currently offered, they realized that their initial idea was similar to an existing program at their organization. As a result, this team revisited their initial program idea and formed a new partnership with the municipal recreation center to address the service gap they identified. This marked the first time that their organization established a formal partnership with an external organization to plan (and potentially implement) a program in their community.

Team 5 had started their community assessment before enrolling in the study and described administering a survey to potential participants and staff. This allowed them to collect information on community needs, interests, and preferences. They also saw these community surveys as a form of *preadvertising* for their future program and used the survey results in drafting their business case.

#### Choose a Program and Cost Implications

Of the 5 teams, 4 (80%) had selected an exercise program before joining the study. These decisions were made based on team members’ knowledge of, and experiences with, existing programs. Team 4 had not selected a program and undertook a comprehensive process to explore the various options, including reading the content in the Planner, exploring program websites and resources, and connecting with exercise program contacts and other teams who had previously used the programs.

Although many (3/5, 60%) of the teams reported that their organization had a set participant enrollment fee, they indicated that the budget planning tool in the Planner helped them to assess what items and resources they had available to them and what unique start-up and ongoing costs they needed to budget for a program designed specifically for people with stroke.

#### Business Case and Implementation Work Plan

In total, 40% (2/5) of the teams completed a business case. Team 3 used the business case template in the Planner and adapted it to suit the needs of their team. Team 5 was required to use their organization’s template to prepare a business case; however, the team was able to use information from the Planner document and steps completed to produce a comprehensive business case in alignment with their usual organizational processes.

The Planner included an implementation work plan template, although only team 4 used it. The reasons given by other teams for not using the implementation work plan included having another preferred method for tracking planning work or not being able to prepare a detailed work plan at this time because of the uncertainty and limitations imposed by the COVID-19–related restrictions.

#### Assess Barriers and Drivers and Address Identified Challenges

Of the 5 teams, 4 (80%) assessed barriers and drivers to program implementation and started planning for how challenges could be addressed. Although this step is described in phase 2, exploring potential barriers tended to occur early and simultaneously with phase 1 activities. In total, 60% (3/5) of the teams engaged with their community planning partners to explore potential challenges, which often resulted in the formation of practical solutions. For example, team 1 held a stakeholder meeting with representatives from local stroke and brain injury groups where they identified concerns related to the location, program time, transportation, and costs. Team 3 had a meeting with their stroke and caregiver planning partners and learned about potential barriers related to the accessibility of the building. This led to changes in their program screening form and development of an information sheet for future participants.

Team 1 proceeded to launch a program but revisited their barriers assessment when they encountered enrollment issues. To understand the factors discouraging people from participating, they continued working with their community partners to identify potential issues with referral pathways, reassess barriers to participation, and consider how these could be addressed in future program sessions.

#### Prepare to Launch and Launch the Program

Because of the pandemic, only 40% (2/5) of the teams worked on this step. Team 3 aimed to complete as much planning work as possible, including launch-readiness activities, with the intention of launching the program when pandemic conditions allowed. However, this site was never able to launch because of ongoing COVID-19–related restrictions. After approximately one year, team 1 was able to launch a session of their exercise program.

#### Develop Evaluation Plan, Monitor Delivery and Use, and Assess Program and Participant Outcomes

Although unable to launch, 40% (2/5) of the teams did spend time exploring how they could evaluate their program. Team 3 prepared a participant satisfaction survey, selected client-centered before-and-after measures, and collaborated with their internal evaluation team to identify available data that could be pulled to show broader system impact. Team 5 reviewed the program-fidelity templates included in the Planner and subsequently selected and adapted one of the tools for use in their setting.

Although at baseline team 1 indicated that the Planner made them think about the importance of developing a comprehensive monitoring plan that includes before-and-after participant outcome measures, at the end of the study the participants explained that they had overlooked this, and it was never implemented. However, they planned to administer a participant satisfaction survey.

#### Evaluate, Adjust, and Sustain

Because of pandemic-related restrictions and delays, none of the enrolled field test teams evaluated, made adjustments, or focused on sustainment during the study period.

### Effects of Using the Planner

#### Participants’ Changing Perceptions of the Planner Itself

As some participants used the Planner, their impressions of the proposed process changed. For example, upon first reviewing the Planner, at least one member from every team expressed concerns about the length and potential complexity of the planning process and tools. However, after gradually working through the planning process over several months during field testing, the participants’ feedback became more positive as they saw the benefits. The following quotes illustrate how a program coordinator changed their views over time as they used the Planner:

There is too much information. It’s overwhelming to read, review, use, and implement.

Program coordinator, team 5, ID12, baseline

Once you get over the size of it, I can’t stress enough what a great resource it is...This [Planner] really gave me a great overview of the right way to plan something...When you see something laid out from start to finish it makes a real big difference. The Planner is going to be so helpful for all programming that I am involved in moving forward. I have learned so much.

Program coordinator, team 5, ID12, end of study

#### Building Capacity for Implementation Planning

All teams gave examples of how the Planner led them to undertake steps and activities that contributed positively to their implementation planning, which they would not have undertaken otherwise. All teams identified ways in which the Planner changed their overall approach to program planning. A participant identified themselves as a *ready-set-go* personality and indicated that the Planner helped them to pause and consider other activities to enhance the success of programs in their organization (eg, conducting a thorough community assessment and developing referral pathways from the community). Another participant identified several Planner steps that were not part of their organization’s usual practices (eg, partner engagement, decision-making methods, and planning for evaluation at the outset) and acknowledged that although these steps would lengthen the planning process, it would be worthwhile. All teams indicated that they would use the Planner for program planning in the future, both to continue planning their current program (when the pandemic-related restrictions allow) and for other program planning. We have summarized the participants’ usual planning process and provided examples of how the Planner changed their implementation-planning knowledge, attitudes, and activities (Table 3).

**Table 3:** Examples of the effects of the Planner on study participants’ implementation-planning knowledge, attitudes, and activities.

Team	Summary of usual approach to planning	Examples of how the Planner influenced planning knowledge, attitudes, and activities	Illustrative quotes from interviews
1	Participants gave differing views: Comprehensive, formalized planning process generally in alignment with the SRiM^a^ Implementation Planner (fitness coordinator) No formal planning framework; experience launching adapted exercise programs (private practice physiotherapist)	Increased knowledge regarding participant-centered considerations (eg, room location) Decision to host a stakeholder meeting to engage community members in the planning process	“We had a stakeholder meeting as a result of utilizing the toolkit. Had I been doing this on my own, I probably would have thought I didn’t need to do that, but it was really good to have. I looked at the Planner before the meeting to think about who do we invite to this meeting? Who are the key stakeholders? What are the key questions we should be discussing at this planning stage? And when we had the stakeholder meeting, it just brought up some really valid points around who are we targeting? Who are we missing? What are the barriers?” [Physiotherapist, team 1, ID1, monitoring interview 1]
2	Program coordinators have significant autonomy to propose and launch new programs. Typically driven by the program of interest and the recreation center, rather than by a formal assessment of needs in the community	More positive attitudes about the benefits of completing early planning steps (eg, partnerships and community assessment) before launching Shifting from an individualized to a more inclusive community-centered planning model	“We’ve been talking about new programs and talking about building relationships with other community partners and the health system, and I’m like, that’s that idea within the Planner—doing that full community survey and getting into the actual community.” [Program coordinator, team 2, ID5, end of study]
3	Programs are typically initiated by staff members within the organization, either as an organizational or provincial directive, or by a frontline staff member seeking managerial approval for a specific program.	Increased ability to use a community-centered approach and successfully engage a diverse team of community stakeholders on the planning team Increased understanding of program planning by working through the Planner	“Just having that [the Planner] as a reference guide for future planning...I think I have a better understanding of how to go about the planning.” [Physiotherapist, team 3, ID6, end of study]
4	Programs to be offered typically built into the job descriptions of clinic staff and based on needs observed in clinic Programs typically planned and implemented in the clinic setting by clinic staff	The Planner process prompted them to shift from a planning team at 1 organization to forming a new partnership with the municipality	“Especially I should say like never working with an outside partner...I’m used to teaching group exercise classes in the hospital, but now we’re looking at doing them outside with groups and partners; it’s uncharted territory for me...Because we are a hospital, a lot of that stuff that the Planner goes through we didn’t have to do because it was already established for us. And now that we’ve decided we are going to be working with the municipality, we’re looking to the Planner even more now for the implementation planning.” [Physiotherapist, team 4, ID10, end of study]
5	Program planning typically driven by an observed community need or through a desire to expand or adapt an existing successful program to other regions	More positive attitudes toward using a formal framework to structure their process Increased knowledge about new steps to integrate into their process (eg, planning for evaluation and fidelity assessment upfront)	“The process has been amazing and it has been really refreshing—we were just rushing to [adapt this program], to now having the process to go oh yeah, let’s use this Planner to direct our focus...we definitely wouldn’t have come to the same place without the Planner.” [Program coordinator, team 5, ID11, end of study]

^a^SRiM: Stroke Recovery in Motion.

### Conditions That Hindered or Facilitated Uptake of the Planner Process by Community Groups

By following the 5 teams and comparing their engagement with the process defined in the Planner, we identified conditions that made the recommended planning elements easier or more difficult to apply in practice. These factors typically related to organizational context and support, team leadership style, the value placed on community-partner engagement, and the COVID-19 pandemic.

#### Challenging Conditions

Teams who followed their organization’s usual processes for planning programs sometimes prematurely judged recommended activities in the Planner as not applicable. Planner use was challenging for organizations and staff primarily focused on the number of programs developed and launched. The comprehensive planning process was viewed as potentially too long to meet usual organizational timelines. The *one-person show* model, in which planning activities are undertaken by 1 organization or person without engagement of a diverse team, similarly discouraged consideration and use of the entire planning process. In addition, organizational contextual factors such as restructuring and changing priorities, as well as broader contextual factors such as the COVID-19 pandemic, created barriers and delays to following the planning process. The COVID-19 pandemic presented significant challenges because organizational priorities and planning timelines shifted quickly and often to address changing pandemic conditions, and difficulties were encountered in forming and maintaining external partnerships.

#### Supportive Conditions

Embracing the Planner process was facilitated by a firm belief in the role of community-based organizations in enhancing the health and well-being of people with stroke. Organizational leaders who valued an evidence-informed planning approach and provided their staff with the dedicated time and resources to work through planning activities created positive conditions supportive of using the Planner. Another positive factor was having a team lead who was open-minded to the Planner process and willing to undertake new steps and activities. Several team leads acknowledged that they personally did not have all the answers or resources and worked early on to identify a diverse group of people to join their core planning team and act as advisers. This openness and engagement led to these teams working through later Planner activities successfully (eg, asking people with stroke and caregivers about their needs and preferences and collaborating with fitness professionals on participant-screening protocols). Finally, for some (2/5, 40%) of the teams, an unanticipated condition that supported Planner use related to the COVID-19 pandemic—with the closure of many services, team members described having *more* time to dedicate to planning.

#### Lessons Learned on How to Effectively Use the Planner

On the basis of their field test experience, participants offered insightful suggestions about how to apply the Planner (Textbox 1), which may be informative to future users.

**Lessons learned from field test participants on how to most effectively use the Stroke Recovery in Motion Implementation Planner.**

**Suggestions for how to use the Planner and illustrative quotes**

- The Planner is a comprehensive document with a lot of information. Get the *big picture* overview, use strategies to break up the content, and flag priority areas for your team.
  - “I was trying to read it all at once when I wasn’t actively doing any planning. Now I am going through it one section at a time and just trying to tackle that section. I’m actively planning as I’m doing it and checking off the tasks, and it definitely feels more manageable.” [Physiotherapist, team 4, ID10, monitoring interview 1]
  - “Even though it’s long, read the whole thing first...If someone’s just starting and they’re going to use the Planner, read it first to keep in mind what you’ve done in the past and then highlight and make notes on the sections that you know are really going to be useful to you. So that way when you do go back you know exactly where to go.” [Program coordinator, team 5, ID12, end of study]
- Do not treat the process as completely linear—give yourself permission to jump around the Planner.
  - “Depending on their personalities, some people are very like ‘I must follow the steps. I must do the step-by-step-by-step’ and I am an example of that person. And so there are some times where it was like well I’ve gotten to here and I haven’t been able to do a proper community survey therefore stop, I cannot go any further because I haven’t done this step yet. Well no, you could still flip forward and see what else you can get started on.” [Program coordinator, team 2, ID5, end of study]
- Take time and get the early stages of the planning process right.
  - “Even if you think you have this all in the bag and you have a program that’s going to start in a couple of months, it’s still really important to go through all of the process because you really need to have those evaluation bits in place from the outset...It’s the depth of quality. Having the time to put into this process will save you time down the road and avoids situations that you can’t really get yourself out of...would that have looked different if we had a different process in place from the beginning?” [Program coordinator, team 5, ID11, end of study]
- Engage with the Planner steps and tools beyond just ticking items off. Take an active approach with the Planner and use it to actually engage with people and organizations.
  - [related to using a Planner tool to assess the facility] “Actually have them go in the building and go through [the checklist] instead of just doing it by memory and what they think it’s going to be. Have someone actually go and do a walk-through of the building.” [Physiotherapist, team 3, ID6, end of study]
- Following the planning process can be more manageable if the load is shared among the team members. Identify a team lead who has the full view of the Planner and can delegate tasks and activities to other team members.
  - “I would have looked at delegating a little bit more. I did a little bit of ‘okay you do this, you do that,’ but I think it could have been done more effectively where [other planning team members] could have taken on some of the bigger pieces...” [Physiotherapist, team 3, ID6, end of study]

## Discussion

### Principal Findings

We followed 5 diverse teams as they used a newly created implementation-planning guide, the Stroke Recovery in Motion Implementation Planner, to plan for the implementation of community-based exercise programs for people with stroke. Because of the COVID-19 pandemic, none of the teams were able to work through the full planning process, with most activities focused on the first of the 3 phases. The findings of this field test study showed that teams took different approaches to applying and adapting the Planner in their settings. All teams indicated that the Planner influenced their approach to program planning and that they intended to continue using the resource in the future. We identified various team, organizational, and broader contextual factors that hindered or facilitated uptake of the Planner by teams. Study participants shared valuable *tips from the field* to help future teams optimize their use of the Planner. Given the paucity of literature reporting implementation toolkit evaluations [30,31], the findings of this study contribute data from our evaluative work on a novel implementation toolkit.

### Comparison With Prior Work

A key finding was that the teams did not complete the planning process in a linear manner. All teams conducted multiple steps simultaneously, with some steps being revisited multiple times during the planning process, a pattern reported elsewhere [16]. We also observed that many planning activities (including those across multiple phases) were worked on early in the planning journey. Although we attributed this finding partly to pandemic-related challenges, previous research found a similar pattern, with teams reporting using an implementation guide most frequently in the early stages [32]. Furthermore, despite all having access to the Planner, no 2 teams followed an identical planning path. Although we are unable to speak to the impact of these diverse planning journeys in terms of implementation outcomes, a previous study using an implementation guide found that it facilitated standardization, while allowing flexibility according to the individual context and resources, with all sites successfully implementing the planned program [33]. Among the barriers to implementation, one of the most common relates to challenges adapting evidence-based interventions [13]. The Planner was therefore designed to be a practical approach to planning based on evidence, which means that it is to be used in alignment with the local context [18]. It was not designed as a *recipe* that must be followed in a lock-step manner. The finding that diverse teams could, in fact, adapt and navigate the planning process in various ways suggests that the resource was applied as intended in real-world settings.

Even experienced planners described new things they learned from the Planner and how this improved their processes, suggesting that the Planner positively influenced end users’ planning capabilities, similar to other work reporting improvements in practitioner implementation skills [34]. In our study, 60% (3/5) of the planning teams were led by, or included, rehabilitation professionals. A recent study [12] of 384 allied health professionals (nearly half of whom were rehabilitation professionals) indicated that these practitioners reported lower levels of confidence in planning, implementation, and evaluation. Nearly all expressed an interest in learning about knowledge translation, with web-based training and resources (such as the Planner) being the preferred format [12].

Fitness professionals are another group essential to planning and implementing fitness programming; yet, it is largely unknown how fitness instructors engage in knowledge translation and implementation and what barriers and facilitators they encounter [35]. Although it is well acknowledged that fitness professionals have a critical role in delivering physical activity interventions and programs, there is limited information on this diverse group’s capacity and training needs [36]. In alignment with the Planner guiding principle of *inclusiveness* [7], we assert that fitness professionals are essential in cocreating the implementation plan. This field test included 12 participants, but only 2 (17%) were fitness professionals, which we attribute to the pandemic-related closure of fitness facilities and layoffs of fitness professionals. The inclusion of only 2 fitness professionals in our study was insufficient to reflect the overall heterogeneity of this group related to education, qualifications, and practice settings. Understanding this diversity and its effect on Planner use by this group may be important because there is evidence that fitness trainers with higher levels of education are more likely to access scientific journals than those with lower education, who prefer mass media and the internet [35]. Future work is needed to understand the implementation capacity and training needs of fitness professionals to enhance full participation in implementation planning.

In this study we used a passive form of implementation; that is, the teams had access to the Planner guide and tools, but no active implementation facilitation or support or any financial incentives were provided. However, there is evidence to suggest that active facilitation can enhance implementation efforts [37,38]. Given the potential value of implementation facilitation, our research team formed a new partnership with March of Dimes Canada, with the goal of having the Planner endorsed and supported by a well-connected and reputable organization in community-based stroke care. March of Dimes Canada has a national After Stroke program [39] that includes regional coordinators, providing the infrastructure to develop its organizational role in implementation facilitation by supporting sites using the Planner. There is an opportunity for future exploratory work that can contribute to the implementation literature on the role of a national organization and its regional coordinators in disseminating and supporting the use of the Planner. Furthermore, although the purpose of this study was not to assess the impact of the Planner on program outcomes, previous literature has reported that implementation toolkits may contribute to improved clinical outcomes [30]; future work is needed to evaluate the influence of the Planner on program outcomes and sustainability.

We identified several cross-cutting barriers and facilitators to following the implementation-planning process proposed in the guide, including organizational context and support, team leadership style, and the value placed on community-partner engagement. These factors were also identified in a review as core capacity–building domains (leadership, organizational climate and culture, partnerships, workforce development, and financial processes) that can be modified to build capacity for the implementation of evidence-based practices [40]. Knowing that simply creating an implementation guide does not guarantee use, we have carefully considered the barriers and facilitators identified in the evaluation of the Planner and have used this information to make changes to the Planner itself. For instance, related to community-partnership engagement, we added new content to the Planner on partnership agreements and a sample invitation letter for stroke and caregiver partners [7]. Furthermore, to address the potential barrier of perceiving that some Planner activities were not applicable, we added “Why is this important” statements throughout to clearly show the potential benefits of completing the various steps, activities, and tools [7].

Finally, it is noteworthy that 80% (4/5) of the teams had already selected the specific exercise program they wanted to implement before enrolling in the study. The finding that most teams had already selected a program speaks to the reality that for many teams, identifying a program is, in fact, the starting point that launches their implementation-planning journey. Observing that not every team starts with a *blank slate* at step 1, we revised the Planner to illustrate various starting points, with directions on how to use the Planner accordingly [7].

### Limitations and Strengths

The greatest limitation of this study was conducting a prospective field test during a pandemic. The pandemic created significant recruitment challenges, and we were unable to recruit our originally planned sample size. Furthermore, for the teams we did enroll in the field test study, the closure of community facilities and the public health restrictions led to significant planning delays. The enrolled teams were therefore unable to progress through the entire planning process, with only 20% (1/5) of the teams able to launch a program during the study period. The purpose of this study was not to ascertain the effectiveness of the Planner related to program implementation and outcomes; rather, we sought to understand if, how, and why the Planner was used in practice. Despite the pandemic-related planning challenges, many teams were still able to work through many steps and provided valuable insight on how they used the Planner, allowing us to meet the field test study objectives. However, it is important to acknowledge that because the teams engaged much more heavily in the early phases and activities of the Planner, we do not have the same depth of understanding of how teams would use the Planner in the later stages (ie, program launch, evaluation, and sustainability).

Furthermore, most (4/5, 80%) of the teams indicated that it was the study itself that prompted them to officially launch their planning activities. The study data collection procedures may have caused participants to engage more with the Planner (eg, completing Planner activities in anticipation of an upcoming monitoring interview with the study team). However, to model more typical program-planning scenarios, the research team did not provide any program funding and provided only minimal assistance with connecting study participants with resources (eg, people and websites). The interviewer (J Reszel) did not contribute to making decisions in the program-planning process. Finally, it is important to note that the monitoring calls and end-of-study interview were completed with 1 primary contact at each site, and their perceptions and experience of working through the Planner may not represent the experiences of their team members.

A strength of the study was the diversity of the sample, which included planning teams with differing compositions based in both urban and rural settings across the country. This allowed us to observe the use of the Planner in a variety of settings, which enhances the transferability of the findings. In addition, despite the challenging pandemic conditions, we were able to maintain contact with 80% (4/5) of the teams throughout the study period, allowing us to collect comprehensive data on Planner use and experiences over time.

### Conclusions

Catalyzing the expansion of safe, effective, and sustainable community-based exercise programs is important to the long-term health of people with stroke. The Stroke Recovery in Motion Implementation Planner [14] is an adaptable resource that may be used in diverse settings to plan and implement community-based exercise programs for people with stroke. The results of this study contribute to the implementation science literature by describing how end users made use of an implementation guide and the influence of the guide on implementation-planning knowledge, attitudes, and activities. These findings may be informative to others who are developing resources to build the capacity of those working in community-based settings to implement new programs and practices. Future work is needed to understand how teams use the Planner to launch, evaluate, and sustain programs; to monitor ongoing use; and to understand the effect and outcomes of using the Planner. The Planner is now hosted by March of Dimes Canada and can be accessed on the organization’s website [14].

## Appendix

Multimedia Appendix 1 Reporting checklist.

Multimedia Appendix 2 Semistructured interview and focus group guides.

Multimedia Appendix 3 Monitoring call documentation form.
