# Supplementary material for: How Community-Based Teams Use the Stroke Recovery in Motion Implementation Planner: Longitudinal Qualitative Field Test Study
Source: JMIR Form Res. 2022 Jul 29;6(7):e37243. doi: 10.2196/37243 (PMC9377454; doi:10.2196/37243)
Supplement: Multimedia Appendix 1 [file formative_v6i7e37243_app1.pdf]

Multimedia Appendix – Completed reporting checklist

This is a Multimedia Appendix to a full manuscript published in the JMIR Form Res. For full copyright and citation information see http://dx.doi.org/10.2196/37243

Consolidated criteria for reporting qualitative research (COREQ) [25]

	Item #	COREQ Item and Guide questions / description	Reported on page #
**Domain 1: Research team and reflexivity**			
Personal characteristics	1	Interviewer/facilitator: Which author/s conducted the interview or focus group?	p. 6
	2	Credentials: What were the researcher's credentials? *e.g. PhD, MD*	p. 6
	3	Occupation: What was their occupation at the time of the study?	p. 6
	4	Gender: Was the researcher male or female?	p. 6
	5	Experience and training: What experience or training did the researcher have?	p. 6
Relationship with participants	6	Relationship established: Was a relationship established prior to study commencement?	p. 6
	7	Participant knowledge of the interviewer: What did the participants know about the researcher? *e.g. personal goals, reasons for doing the research*	p. 6
	8	Interviewer characteristics: What characteristics were reported about the interviewer/facilitator? *e.g. Bias, assumptions, reasons and interests in the research topic*	p. 6
**Domain 2: Study design**			
Theoretical framework	9	Methodological orientation and theory: What methodological orientation was stated to underpin the study? e.g. grounded theory, discourse analysis, ethnography, phenomenology, content analysis	p. 4;<br>p. 6
Participant selection	10	Sampling: How were participants selected? *e.g. purposive, convenience, consecutive, snowball*	pp. 4-5
	11	Method of approach: How were participants approached? *e.g. face-to-face, telephone, mail, email*	pp. 4-5
	12	Sample size: How many participants were in the study?	p. 10
	13	Non-participation: How many people refused to participate or dropped out? Reasons?	p. 10
Setting	14	Setting of data collection: Where was the data collected? *e.g. home, clinic, workplace*	p. 6
	15	Presence of non-participants: Was anyone else present besides the participants and researchers?	p. 6
	16	Description of sample: What are the important characteristics of the sample? *e.g. demographic data, date*	Table 1<br>Table 2
Data collection	17	Interview guide: Were questions, prompts, guides provided by the authors? Was it pilot tested?	pp. 5-6;<br>Appendix 2
	18	Repeat interviews: Were repeat interviews carried out? If yes, how many?	pp. 5;<br>Table 2
	19	Audio/visual recording: Did the research use audio or visual recording to collect the data?	p. 6
	20	Field notes: Were field notes made during and/or after the interview or focus group?	p. 6
	21	Duration: What was the duration of the interviews or focus group?	p. 10
	22	Data saturation: Was data saturation discussed?	-
	23	Transcripts returned: Were transcripts returned to participants for comment and/or correction?	p. 5-6
**Domain 3: Analysis and findings**			
Data analysis	24	Number of data coders: How many data coders coded the data?	p. 7
	25	Description of the coding tree: Did authors provide a description of the coding tree?	pp. 6-7

	Item #	COREQ Item and Guide questions / description	Reported on page #
	26	Derivation of themes: Were themes identified in advance or derived from the data?	pp. 6-7
	27	Software: What software, if applicable, was used to manage the data?	p. 7
	28	Participant checking: Did participants provide feedback on the findings?	p. 5
Reporting	29	Quotations presented: Were participant quotations presented to illustrate the themes / findings? Was each quotation identified? *e.g. participant number*	pp. 11-24; Table 3 Table 4
	30	Data and findings consistent: Was there consistency between the data presented and the findings?	pp. 11-24; Table 3 Table 4
	31	Clarity of major themes: Were major themes clearly presented in the findings?	pp. 11-24
	32	Clarity of minor themes: Is there a description of diverse cases or discussion of minor themes?	pp. 11-24
