# Supplementary material for: How Community-Based Teams Use the Stroke Recovery in Motion Implementation Planner: Longitudinal Qualitative Field Test Study
Source: JMIR Form Res. 2022 Jul 29;6(7):e37243. doi: 10.2196/37243 (PMC9377454; doi:10.2196/37243)
Supplement: Multimedia Appendix 2 [file formative_v6i7e37243_app2.pdf]

Multimedia Appendix – Semi-Structured Interview/Focus Group Guides

This is a Multimedia Appendix to a full manuscript published in the JMIR Form Res. For full copyright and citation information see http://dx.doi.org/10.2196/37243

Baseline questions

1. Let's start by talking about usual processes for planning programs at your organization.
   - What is the usual planning process? Is there a formal framework or process that you follow?
     - Who initiates program planning at your organization?
     - What is the motivation - key factors/key moment - that triggers contemplation of a new program?
     - How do you set priorities? How are the decisions made? Who makes the decisions? What is the approval process? How do you allocate limited resources?
     - Which populations need to be included to create critical mass? (i.e., how many participants do you need to move forward? How did you decide?)

2. Now let's talk more specifically about implementing a community-based exercise program for people living with stroke.
   - Tell me how it came about that your community started thinking about implementing a community-based exercise program for people living with stroke?
   - Had any planning work been done before you enrolled in this study? If yes:
     - Tell me about what work had been done. Walk me through the steps you took.
       - When did your planning start?
       - Who was involved?
       - Are there any previously completed planning documents you could share with us?
     - How did the steps you previously took compare to the Planner?
       - If completed a step in the Planner before study enrollment – how close is what we suggested to what you actually did?
       - Did you take any steps not covered in the Planner? Did we miss anything?
     - If you had access to the Planner before, would you have approached things differently?

3. Let's now turn to the Planner and its tools and your initial impressions.
   - Could you start by telling me your initial reaction to the planning process described in the Planner?
     - Does it make sense to you?
     - Are the phases the right phases?
     - Are the steps the right steps?
   - How similar or dissimilar is the process to your current planning process?
   - How feasible do you think the process will be to follow?
   - What do you think will be the challenges of following the Planner? How might you overcome those challenges?
   - What do you think of the Planner in general?
     - What do you like or dislike about it?
     - How complete is it?

  - What do you think of the tools?

4. Now let's talk about some of the recommended modifications to the Planner and tools.
   [If participant completed survey]
   - Thank you for completing the survey. I have looked over your responses and want to talk more in depth about some of your feedback. Let's talk about some of the specific items that you recommended should be changed or removed in your survey.
     - You identified that section(s) X needed modification….[probe as needed]
     - You identified hat section(s) X should be removed…..[probe as needed]

5. Now let's talk about your planning team – including how it was formed and how you are working together at this point.
   - Tell me who is on the planning team?
   - Who would be the person(s) responsible for leading program planning (local champion)?
   - How did you become engaged with this planning team?
   - Tell me about your role and responsibilities on the team.
   - As you have been building the planning team, what has been working well to engage the team members? Have you encountered any challenges to date?
   - Who else have you identified as key stakeholders? What do you anticipate the role of these other stakeholders will be?
     i. Examples might include: any other people in the community with an interest in the success of the program, funders, people living with stroke, delivery personnel, people in the referral network, caregivers, etc.

6. Other comments
   - Do you have any other comments on the Planner?
   - Do you have any other comments on your team's plans for implementing a community-based exercise program for people living with stroke?

Monitoring questions

1. Tell me about what your team has worked on since we last talked.

2. Have you been referring to the planner? If so, which sections have you been working through? How did it go?

3. Have you completed any of the tools? If so, who completed them? If so, can we have a copy of completed tools?

4. Did you use any other tools or resources? If so, what were they? Is this something we should consider adding to the planner?

5. What is your key issue / most pressing need / biggest challenge right now? Do you need help with anything? Are you getting the help you need?

6. Have there been any positive developments since we last spoke? Biggest success? What is helping the most?

7. What are your next steps?

End-of-study questions

1. How did using the Planner contribute to your experience planning a community-based exercise program?

2. Reflecting over your past few months using the Planner:
   - o What parts of the Planner were the most helpful?
   - o Are there steps you followed but in hindsight don't think were helpful?
   - o Is there anything you felt was missing from the Planner?

3. While the formal SRiM study is ending, we are wondering if you will continue to use the Planner as you continue your work to plan and deliver a community-based program for people living with stroke? Why or why not.

4. Would you recommend the Planner to others? If so, who?

5. What would be your advice for another team on how to use the Planner?

6. During our previous calls, you indicated that you completed [*names of SRiM tools*] and/or used your own tools [*names of own tools*]. As I had mentioned at the start of the study, we are interested in collecting these documents from our participating sites so we can learn how real-world teams use the Planner tools and other resources. Would you be willing to share your completed tools with us? You could remove any identifying information before sending them to me.

7. Is there anything else you wanted to share?
