# Supplementary material for: How Community-Based Teams Use the Stroke Recovery in Motion Implementation Planner: Longitudinal Qualitative Field Test Study
Source: JMIR Form Res. 2022 Jul 29;6(7):e37243. doi: 10.2196/37243 (PMC9377454; doi:10.2196/37243)
Supplement: Multimedia Appendix 3 [file formative_v6i7e37243_app3.pdf]

MONITORING CALL DOCUMENTATION FORM

This is a Multimedia Appendix to a full manuscript published in the JMIR Form Res. For full copyright and citation information see http://dx.doi.org/10.2196/37243

**Participant ID** *(as per master list)*	Click here enter text.
**Monitoring contact date**	Click here to enter a date.
**Name of research staff**	Click here enter text.
**Method of contact**	Click here enter text.
**Planned date of next monitoring call**	Click here to enter a date.

**Tasks/Activities discussed during this call**<br><br>*(check all that apply)*	**PHASE 1**<br>☐ A1 Explore the call to action<br>☐ A2 Involve the community<br>☐ A3 Importance of exercise for people with stroke<br>☐ A4 Conduct a community assessment<br>☐ A5 Choose a program + cost implications<br>☐ A6 Business case and implementation work plan<br>☐ A7 Proceed or not<br>**PHASE 2**<br>☐ B Assess barriers and drivers<br>☐ C Address identified challenges<br>**PHASE 3**<br>☐ D Launch the program<br>☐ E Develop evaluation plan; monitor delivery and use<br>☐ F Assess participant and program outcomes<br>☐ G Continued operation of program
**Tools completed / or data used discussed during this call**<br><br>*(check all that apply)*	☐ Implementation Planning Roadmap<br>☐ Project Charter: Template<br>☐ Declaration of Conflict of Interest: Sample Disclosure<br>☐ Community (Environmental) Scan: Worksheet<br>☐ Community/Environmental Readiness: Worksheet<br>☐ Sample Questions to Assess Community Readiness<br>☐ Feasibility, Applicability, and Acceptability<br>☐ Budget Planning: Worksheet<br>☐ Preparing the Business Case: Template<br>☐ Implementation Workplan: Template<br>☐ Identification of Barriers and Drivers: Template for Solution Building<br>☐ Medical Authorization: Fit for Function Program Sample<br>☐ Recreation Centre Readiness Checklist: TIME Program Sample<br>☐ Participant Fitness Progress Log: Fit for Function excerpt<br>☐ Program Sustainability Assessment Tool (PSAT)<br>☐ Other – specify:
**Upcoming plans**	

Tell me about what your team has worked on since we last talked.
•

Have you been referring to the planner? If so, which sections have you been working through? How did it go?
•

Have you completed any of the tools? Who completed them? If so, can we have a copy of completed tools?
•

Did you use any other tools or resources? If so, what were they? Is this something we should consider adding to the planner?
•

What is your key issue / most pressing need / biggest challenge right now? Do you need help with anything? Are you getting the help you need? Anything we can do?
•

Have there been any positive developments since we last spoke? Biggest success? What is helping the most?
•

What are your next steps?
•

When should we plan to talk next?
•

Phase No. (1-3)	Task letter (A-G)	Detailed notes on key questions from process tracker by task/activity
		•
		•
		•
		•
		•
		•

Other notes:
•
